# Phenotype Characterization of a Mice Genetic Model of Absolute Blindness

**DOI:** 10.3390/ijms23158152

**Published:** 2022-07-24

**Authors:** Santiago Milla-Navarro, Mateo Pazo-González, Francisco Germain, Pedro de la Villa

**Affiliations:** 1 Department of Systems Biology, University of Alcala, 28801 Madrid, Spain; santiago.milla@edu.uah.es (S.M.-N.); mateo.pazo@edu.uah.es (M.P.-G.); 2 Visual Neurophysiology Group-IRYCIS, 28034 Madrid, Spain

**Keywords:** murine model, blindness, photosensitivity, *Opn4* ^−/−^, *Pde6b^rd10/rd10^*

## Abstract

Recent technological development requires new approaches to address the problem of blindness. Such approaches need to be able to ensure that no cells with photosensitive capability remain in the retina. The presented model, *Opn4*^−/−^ × *Pde6b*^rd10/rd10^ (O×Rd) double mutant murine, is a combination of a mutation in the *Pde6b* gene (photoreceptor degeneration) together with a deletion of the *Opn4* gene (responsible for the expression of melanopsin in the intrinsically photosensitive retinal ganglion cells). This model has been characterized and compared with those of WT mice and murine animal models displaying both mutations separately. A total loss of pupillary reflex was observed. Likewise, behavioral tests demonstrated loss of rejection to illuminated spaces and a complete decrease in visual acuity (optomotor test). Functional recordings showed an absolute disappearance of various wave components of the full-field and pattern electroretinogram (fERG, pERG). Likewise, visual evoked potential (VEP) could not be recorded. Immunohistochemical staining showed marked degeneration of the outer retinal layers and the absence of melanopsin staining. The combination of both mutations has generated an animal model that does not show any photosensitive element in its retina. This model is a potential tool for the study of new ophthalmological approaches such as optosensitive agents.

## 1. Introduction

Currently, there are a large number of animal models that show alterations in the visual system similar to those present in human diseases [1,2,3]. These models are useful to study the pathophysiology of these diseases and allow the development of new fields of study such as photopharmacology [4,5,6]. These new therapies aim to apply drugs that are activated by light stimulation to achieve a certain effect. Many potential applications have been raised, such as for cancer, chronic pain, central nervous system pathologies, diabetes, or blindness. One of its advantages is that only those targets where it should take effect are stimulated, avoiding or reducing side effects. In the case of blindness, the implantation of a molecule in the retina that would cause a signal to be transmitted to the rest of the visual pathway when stimulated by light could restore functionality to that pathway. In order to test different molecules, animal models of absolute blindness are needed, since any cell with photosensitive capacity that remains in the retina could cause a bias in the interpretation of the results after the application of these photosensitive molecules [7,8].

There is a large number of animal models that allow studying the progression of diseases that occur with the loss of the structure and functionality of the various cell layers that make up the retina [1,2]. However, not all currently existing animal models are valid for achieving this goal. It is necessary that the total elimination of the photosensitive capacity of the retina takes place, not only of the classical receptors, but also of the intrinsically photosensitive ganglion cells (ipRGCs) [9,10,11]. The *Pde6b^rd10/rd10^* (Rd10) mice are a very well-characterized animal model, corresponding to retinitis pigmentosa. They present a mutation at the C-terminal end of the phosphodiesterase 6b (*Pde6b)* gene, specifically in exon 13 of this gene, which encodes the β subunit of rod phosphodiesterase [3,12]. This truncated subunit prevents the hydrolysis of cGMP from taking place inside the cell, so the cGMP-dependent channels cannot be closed and the influx of cations into the membrane does not stop, finally activating the death pathway in photoreceptors [13]. This degeneration is similar to that which occurs in some cases of retinitis pigmentosa in humans, causing the disappearance of the rods initially, and later that of the cones. However, these animals maintain the structure and functionality of the inner retina [1,13,14,15]. All this makes this animal model a good tool for the study of this disease, as well as for the application of new therapeutic tools [16,17,18,19,20].

However, the Rd10 model had an important limitation: in several studies with animal models of rod and cone degeneration, behaviors or physiological processes associated with photosensitivity were recorded [21,22,23]. In this way, it was found that there were other cells, the ipRGCs, which were involved in a series of processes associated with photosensitivity, but were not part of the imaging processes, as rods or cones were. Processes such as the regulation of circadian cycles or the pupillary reflex are largely influenced by these photosensitive cells, among others [24,25,26,27]. This type of ganglion cell owes its photoreceptor capacity to melanopsin, encoded by the *Opn4* gene, a protein belonging to the opsin family that endows these ganglion cells with photosensitive activity [28]. Opsins are related to a series of physiological processes that are not involved in image formation, such as the pupillary reflex [28] and circadian cycles [29,30,31], among others. A *Opn4* gene deletion will prevent the expression and synthesis of the photosensitive pigment of inherently photosensitive ganglion cells and melanopsin [9,11,32].

The *Opn4^−/−^*× Rd10 animal model is characterized by the combination of two genetic mutations that lead to the loss of all photosensitive elements of the retina, from 60 days postnatally. The use of an animal model, which does not present any type of photosensitive element in its retina, could be a great asset when trying new avenues of research focused on the restoration of visual functions, such as in optogenetics or optopharmacology.

In this work, an animal model of absolute blindness has been exhaustively characterized, generated by crossing mice with photoreceptor degeneration (Rd10) and mice that present a mutation that inhibits the synthesis of melanopsin (*Opn4^−/−^*). The objective is to verify that the *Opn4^−/−^* × Rd10 (O×Rd) model meets the necessary requirements to consider its blindness as absolute. For this, immunohistochemical techniques were performed to demonstrate the presence or absence of the photoreceptors involved; electrophysiological tests that characterized the function of the different types of photoreceptors by measuring the amplitude of the waves that are generated in the retina and in the visual cortex; measurements of visual acuity; and finally, behavioral tests as a final expression of said ability.

## 2. Results

### 2.1. Light/Dark Transition Test

**Figure 1:**
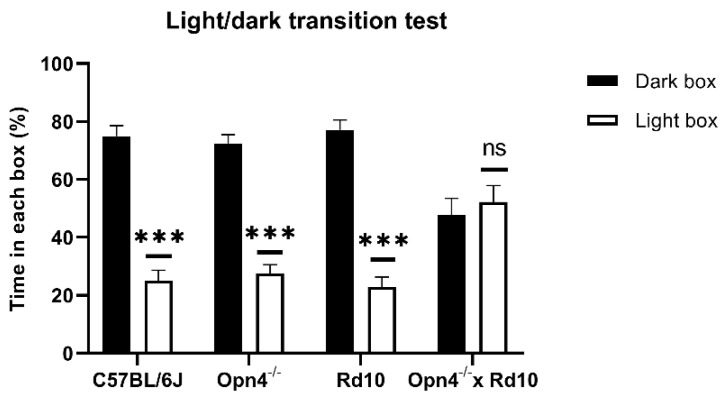
Percentage of time spent by each animal model (C57BL/6J, *n* = 8; *Opn4^−/−^ n* = 8; Rd10, *n* = 8; *Opn4^−/−^* × Rd10, *n* = 8), in each of the compartments of the light/dark transition test. Histograms in black correspond to time spent in the dark compartment, and white histograms, the time spent in the illuminated compartment. The *Opn4^−/−^* × Rd10 animal model is not capable of differentiating between the two compartments, unlike the rest of the animal models, which have longer stays in the dark compartment. Data are presented as the mean ± SEM. (***, *p* < 0,001; ns, not significant).

As can be seen in Figure 1, the animals that presented some type of photosensitive element in the retina spent a greater percentage of the time of the experiment in the dark chamber, with brief visits to the illuminated chamber. This can be clearly observed in C57 animals (*n* = 8), whose retinas were in perfect condition. These animals were kept approximately 75% of the time in the dark compartment, showing a strong rejection of the illuminated compartment, which they only accessed sporadically, driven by their exploratory sense. In the same way, *Opn4^−/−^* animals (*n* = 8), whose melanopsin cells are unable to express the photosensitive pigment, remained in the dark compartment for a longer time, since they still maintained the classic photoreceptor cells (rods and cones). Surprisingly, the Rd10 animals (*n* = 8) showed the same rejection towards the illuminated compartment as the C57 and *Opn4^−/−^* models, staying in the dark compartment for a higher percentage of the time. This suggests that melanopsin cells allow for the detection of high luminosity stimuli, even when the centripetal transmission path of the retina has been compromised.

Unlike the rest of animal models, the O×Rd double mutants (*n* = 8), which do not have any type of photosensitive element in their retinas, are therefore not capable of differentiating between both compartments, freely exploring both equally, remaining approximately 50% of the time in each compartment.

No statistically significant differences were detected in the percentage of time spent in the two compartments between any of the animal models that presented some photosensitive element in their retinas (Two-Way ANOVA, *p* > 0.05). However, there are significant differences (Two-Way ANOVA, *p* < 0.0001) between the three light-sensitive groups and the O×Rd double mutants.

### 2.2. Optomotor Test

**Figure 2:**
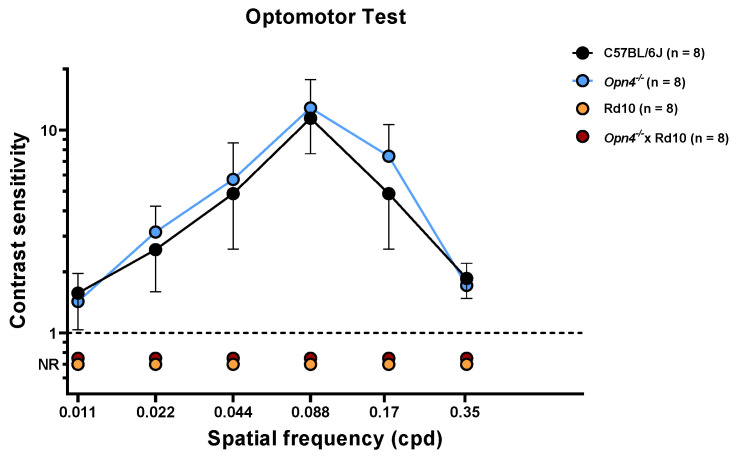
Evaluation of visual acuity of the different animal models using the optomotor test. (C57BL/6J (●), *n* = 8; *Opn4^−/−^* (●), *n* = 8; Rd10 (●), *n* = 8; *Opn4^−/−^* × Rd10 (●), *n* = 8). While the *Opn4^−/−^* animal model did not show any difference in contrast sensitivity compared to the control model, the animal models that presented photoreceptor degeneration (Rd10 and *Opn4^−/−^* × Rd10) were not able to respond to any presented stimulus (*p* < 0,001). Data are presented as mean ± SD.

This test measures the visual acuity of the animals, observing the movement that the animal makes with its head when following the direction of rotation of the bar stimuli. The spatial frequency of the stimuli, direction, and contrast of these were varied in order to determine visual acuity. As can be seen in the data shown in Figure 2, the C57 (*n* = 8) and *Opn4^−/−^* (*n* = 8) animal models show high visual acuity.

The sensitivity of these animals is maximum for a spatial frequency of 0.008 cpd and decreases progressively and symmetrically as the spatial frequency increases or decreases. This sensitivity is drastically reduced, and equally, at the highest and lowest spatial frequencies. No statistically significant differences were observed between the experimental groups C57 and *Opn4^−/−^* (two-way ANOVA, *p*-value > 0.05). This suggests that both animal models present a retinal circuitry capable of discerning between the different types of stimuli. The absence of melanopsin cells in the *Opn4^−/−^* model does not seem to negatively affect the visual acuity of these animals. On the contrary, the animals that have suffered the degeneration of the photoreceptors, (Rd10 (*n* = 8) and O×Rd (*n* = 8)), are not able to follow the direction of rotation of the different stimuli used, so they cannot give any answer. The differences between the animals that do not suffer degenerative processes (C57 and *Opn4^−/−^*) and the models with retinal degeneration (Rd10 and O×Rd) are extremely significant (two-way ANOVA, *p* < 0.0001).

As with the light/dark transition test, the O×Rd double mutant animals do not present any type of behavioral variation in the presence of visual stimuli presented in their environment.

### 2.3. Pupillary Light Reflex

**Figure 3:**
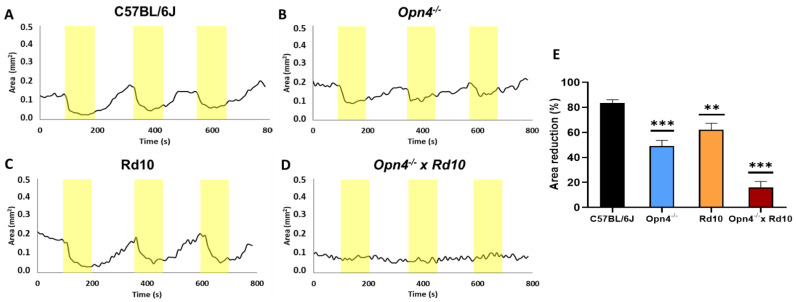
Variation of the pupillary area of the different animal models: C57BL/6J, (**A**); *Opn4^−/−^*, (**B**); Rd10, (**C**); and *Opn4^−/−^* × Rd10, (**D**), after a light stimulus. Representation of the percentage of pupillary contraction of the different animal models (*n* = 8 for each group) when exposed to light stimuli (yellow stripes). The models with the mutation in the melanopsin gene (*Opn4^−/−^*), showed a significant decrease in the pupillary contraction capacity (**E**). The double mutant animals did not show any sign of pupillary contraction when exposed to light stimuli. The area was measured in mm^2^. Data are presented as mean ± SEM, (**, *p* < 0.005; ***, *p* < 0.001).

The physiological pupillary reflex is observed at C57, being the greatest contraction of the pupil in response to the light stimulus. The rest of the models showed a significantly lower contraction. Within the experimental models, the O×Rd model (*n* = 8) presented a percentage of variation of the pupillary area much lower than all the other groups (Figure 3).

This analysis confirms that, although both classical photoreceptors and melanopsinic RGC contribute to the pupillary reflex, it rests fundamentally on the latter. The data that suggest this derive from the fact that compared to C57, *Opn4^−/−^* animals reduce their pupillary contraction capacity by more than 30% (t-Student, *p* < 0.0001), while Rd10 do so by only 20% (t-Student, *p* = 0.0018), despite the fact that the retinal circuitry is much more affected in the Rd10 model, due to the loss of rods and cones.

Unlike the other models, the O×Rd double mutant hardly exhibits the pupillary contraction capacity to light stimuli, losing approximately 70% of the contraction capacity of C57 animals (t-Student, *p* < 0.0001).

### 2.4. Full-Field Elecroretinography

**Figure 4:**
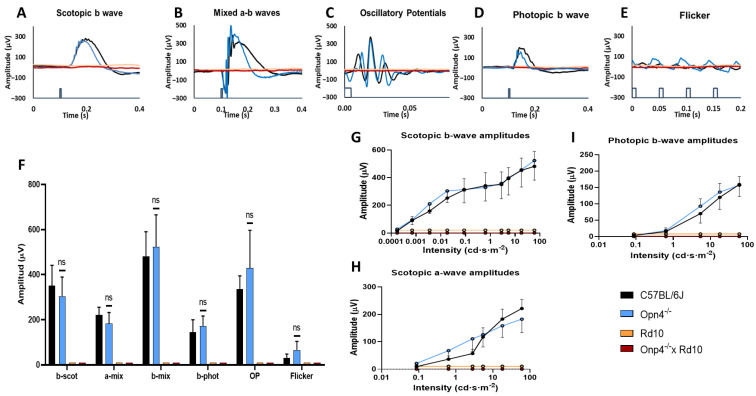
ERG waves in the models Rd10, *Opn4^−/−^* × Rd10, C57BL/6J, and *Opn4^−/−^*. Rod waves are shown (**A**), mix response (**B**), oscillatory potentials (**C**), cones response (**D**), flicker (**E**). Histogram of response amplitude for b scotopic waves, a mixed, b mixed, b photopic, oscillatory potentials, and flicker (**F**), and the illumination intensity–amplitude response curves for scotopic b waves (**G**), b photopic (**I**), and a scotopic wave (**H**). The sample size was eight mice for all groups. While the *Opn4^−/−^* animal model does not show significant differences compared to the control, the models that present photoreceptor degeneration (rd) were hardly able to evoke any type of electrophysiological response from the cellular elements of the retina (*p* < 0.001). Data are presented as the mean ± SD. (ns, not significant).

This test analyzes the functional response of the different elements of the retina to light stimulation. It was observed that the Rd10 (*n* = 8) and O×Rd (*n* = 8) models showed in their recordings a total loss of the different components of the ERG waves, while the C57 (*n* = 8) and *Opn4^−/−^* (*n* = 8) maintained their characteristic components (Figure 4A–E). These differences were statistically significant (two-way ANOVA, *p* < 0.0001) (Figure 4F).

In contrast, between the C57 and *Opn4^−/−^* models, no significant differences were observed (two-way ANOVA, *p* = 0.3110) in the amplitude of any of the ERG waves after illumination. Examples of wave b (Figure 4G) under dark adaptation conditions are shown (two-way ANOVA, *p* = 0.2662); wave a (Figure 4H) (two-way ANOVA, *p* = 0.3305); and the photopic b wave (Figure 4I) (two-way ANOVA, *p* = 0.2301). However, the differences of C57 and *Opn4^−/−^* with the other experimental groups (Rd10 and O×Rd) were extremely significant (two-way ANOVA, *p* < 0.0001).

### 2.5. Pattern Elecroretinography

This test studies the functional state of the innermost layers of the retina by analyzing their response to different spatial frequencies. To carry out this study, the *Opn4^−/−^* and Rd10 groups were dispensed with, since the previous data showed that there were no significant electroretinographic differences between the *Opn4^−/−^* and C57 groups, nor between the Rd10 groups and the O×Rd double mutant.

**Figure 5:**
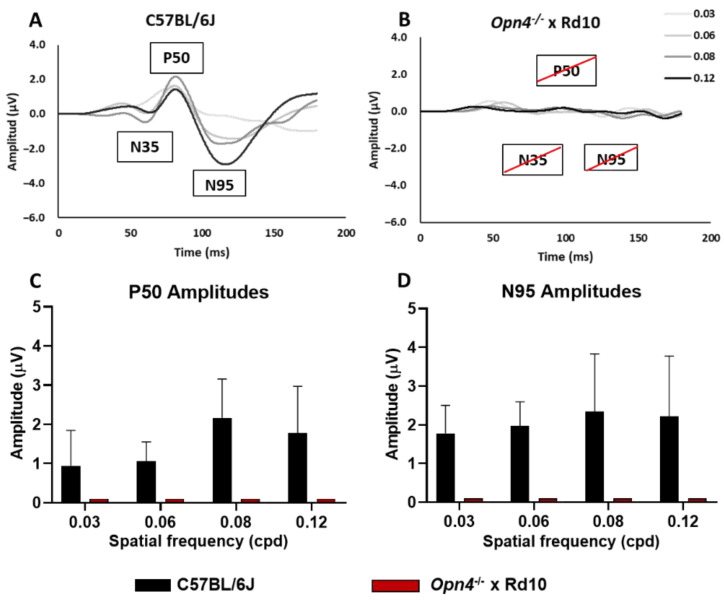
Pattern electroretinogram. Record of the waves corresponding to the strains C57BL/6J (**A**), and *Opn4*^−/−^ × Rd10 (**B**). Wave amplitude histograms P50 (**C**) and N95 (**D**). There are marked differences in the model *Opn4*^−/−^ × Rd10, compared to control (*p* < 0.001). Data are presented as the mean ± SD.

The animals of the C57 (A) group (*n* = 8) clearly showed the characteristic components of the pERG waves, while those of the O×Rd experimental group were not able to evoke any of the three characteristic components (N35, P50, N95) of pERG waves visible in recordings of C57 animals. Thus, the differences between both groups were extremely significant for the amplitude of their components. Statistical analysis showed significant differences (two-way ANOVA, *p* < 0.0001) between both groups (Figure 5C,D).

### 2.6. Visual Evoked Potentials

This analysis, by recording the response of the visual cortex to light stimulation, makes it possible to check the integrity of the retina pathways and central nervous system nuclei to such stimulation.

**Figure 6:**
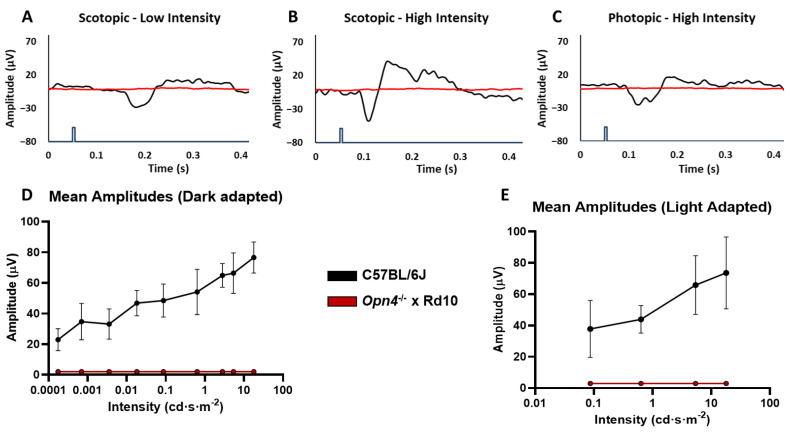
Visual evoked potentials (VEP). Record of the waves corresponding to the strains C57BL/6J and *Opn4*^−/−^ × Rd10 in a dark adaptation situation with low light intensity (**A**), dark adaptation with high light intensity (**B**), light adaptation with high light intensity (**C**). Lighting intensity curves–response amplitude in dark adaptation situation ((**D**)) and light adaptation situation (**E**). The animal model *Opn4*^−/−^ × Rd10 was not capable of evoking any VEP in the visual cortex in response to light stimuli (*p* < 0.001). The blue line represents the light stimulus. Data is presented as the average ± SD.

While the C57 group showed normal responses to flash-type light stimulation, the experimental O×Rd group was not capable of evoking any type of cortical response, as can be seen in the different recordings (Figure 6A–C).

As with the rest of the electrophysiological tests, a total decrease in the amplitudes of the characteristic components of the VEP (D) is observed in the experimental group O×Rd (*n* = 8), with respect to the group of C57 (*n* = 9) (two-way ANOVA, *p* <0.0001).

The analysis of the amplitude variations as a function of the applied light intensity (Figure 6E) showed that the amplitude of the P2 component increased as a function of the light intensity applied in the C57 animals, while there was no variation in the O×Rd experimental group. Therefore, the comparison between both groups showed very significant differences in both dark adaptation and light adaptation (two-way ANOVA, *p* < 0.0001 in both cases).

### 2.7. Inmunohistochemistry

**Figure 7:**
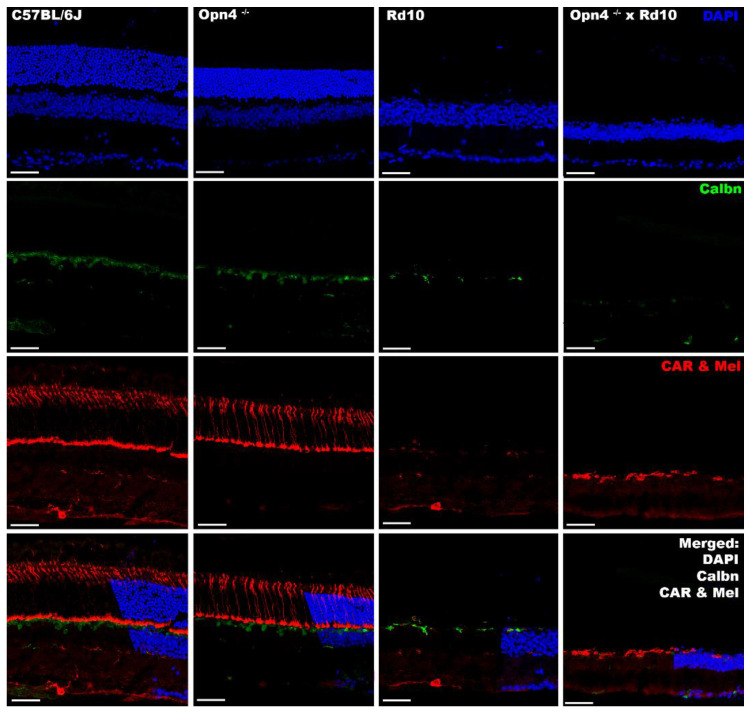
Immunohistochemical labeling of cones and ipRGCs in different models. From left to right, the different animal models are presented (C57BL/6J; *Opn4^−/−^*; Rd10; *Opn4^−/−^* × Rd10). From top to bottom, the different agents and antibodies used are presented. **DAPI**: intercalating marker of cell nuclei; **Calbindin (Calbn)**: protein located in the horizontal cells; **Cone Arrestin (CAR)**: cone labeler; **Melanopsin (Mel)**: melanopsin labeler, located in the ipRGCs; ipRGCs are marked by white arrowhead. The bottom row shows: “**Merged**” of different labelings. *Opn4^−/−^* × Rd10 animal model showed total absence of cells with photosensitive capacities. The scale bar indicates 50 µm.

In the different models studied, cell nuclei and cones were labeled by DAPI and cone arrestin, respectively. It was observed that the animal models of retinal degeneration (Rd10 and *Opn4^−/−^* × Rd10) showed serious damage to the outer layers of the retina (outer segments of the photoreceptors and outer nuclear layer), as they lacked labeling compared to non-degenerate models (C57BL/6J and y *Opn4^−/−^*) (Figure 7).

In these degeneration models, a decrease in the labeling for the calbindin protein is also observed, which fundamentally marks the horizontal cells of the retina. In the animal model, O×Rd disappears completely.

The lack of melanopsin, the photosensitive pigment expressed by inherently photosensitive ganglion cells, was demonstrated in the *Opn4^−/−^* and O×Rd animal models, in contrast to the C57 and Rd10 animal models, where it was present (Figure 7).

**Figure 8:**
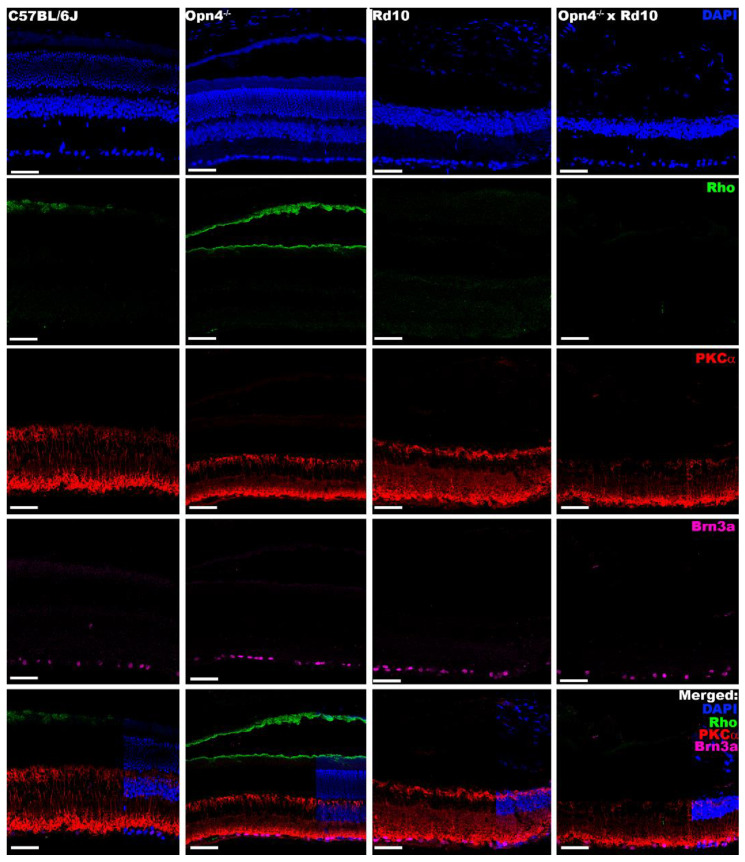
Immunohistochemical labeling of external segment of rods, rod bipolar cells, and RGCs. From left to right, the different animal models are presented (C57BL/6J; *Opn4^−/−^*; Rd10; *Opn4^−/−^* × Rd10). From top to bottom, the different agents and antibodies used are presented. **DAPI**: intercalating marker of cell nuclei; **Rhodopsin (Rho)**: opsin located in the outer segments of the rods**; PKCα**: bipolar cell structural marker; **Brn3a**: protein located in the ganglion cells of the retina. The bottom row shows “**Merged**” of different labelings. As can be seen, in the models that present photoreceptor degeneration (rd), the marking of the outer segments of the photoreceptors disappears, while the cells of the inner retina show no apparent differences in any of the four animal models. The scale bar indicates 50 µm.

On the other hand, a second labeling completed the structural study of the deficiencies in the different models. While in the non-degenerative models (C57 and *Opn4*^−/−^) the outer layers of the retina can be observed, in the degeneration models (Rd10 and O×Rd), its absence is appreciated. In addition to the loss of the soma layer in the outer nuclear layer, the loss of the outer segments of the rods was detected by means of rhodopsin (pigment of the outer segments of the rods) (Figure 8).

For the study of the cells to which the rods communicate their signal, the rod bipolar cells, PKC alpha labeling was used. Unlike the previous results, it was observed that these cells remained in all the models studied (Figure 8). The next scale in the centripetal pathway of the visual pathway, the retinal ganglion cells, likewise, remained in all models, as indicated by the Brn3a labeling (Figure 8). This indicates that the internal retina does not appear to present any type of significant structural alteration.

In summary, immunohistochemically, it can be said that the Rd10 retina lacks the photoreceptors of the outer retina (rods and cones); the retina of *Opn4^−/−^* mutants lacks melanopsinic RGC and that the retina of *Opn4^−/−^* × Rd10 double mutants does not present any cellular components with photosensitive capacity.

## 3. Discussion

Recent technological development has allowed for the emergence of new approaches to address the problem of blindness. Such approaches need to be able to ensure that no cells with photosensitive capability remain in the retina. Until now, few models allowed this objective, especially if a fast degeneration rate was required.

The *Opn4^−/−^* × Rd10 animal model is characterized by the combination of two genetic mutations. On the one hand, a mutation in the *Pde6b* gene, which will lead to the degeneration of rods and cones [13], simulating human pathologies of vision loss such as retinitis pigmentosa [33,34]. On the other hand, the animal has a mutation in the *Opn4* gene, which prevents the expression and synthesis of the photosensitive pigment of intrinsically photosensitive ganglion cells and melanopsin [9,11,32].

This model has been studied from a structural, functional, and behavioral point of view to rule out that the retina had any photosensitive capacity. The intention is that it serve as a model to test different photosensitive molecules that can restore functionality to the visual pathway.

Animal models of classical photoreceptor degeneration have shown to preserves sufficient photosensitive capacities to discern illuminated spaces or even maintain the pupillary reflex [21,22,23,24], due to the presence of melanopsin cells in the retina. This is what prevents the use of rd/rd models to test new molecular tools such as Photoswitches, since they would maintain photoreceptor cells capable of influencing the behavior of animals when faced with lighting, so the effect of photoswitches could not be properly evaluated.

To characterize the different animal models and ensure their absolute blindness, the light/dark transition test, the optomotor Test, and study of pupillary reflex were performed [35,36].

The light/dark transition test is a technique based on the rejection of mice towards open and illuminated spaces [37,38,39,40]. Only the O×Rd double mutant explored both chambers equally, while the rest of the groups showed a significant rejection to the illuminated camera, which demonstrated that they perceived some light.

Animal models lacking classical photoreceptors (rods and cones) have been shown to maintain the pupillary reflex [21,23,24] because photoreceptors collaborate with ipRGCs [41,42]. Classic photoreceptors are related to constriction at low light intensities, while ipRGCs cells are essential at higher intensities, in addition to maintaining pupillary contraction for longer [43]. Since C57BL/6J, *Opn4^−/−^* and Rd10 mice have some of these cells, they are capable of contracting the pupil when they are exposed to a light source. From a quantitative point of view, the greater pupillary contraction of the Rd10 mice compared to the *Opn4^−/−^* mice indicates that the melanopsin cells maintain the main control of the signaling pathway that activates the pupillary reflex. Unlike previous models, the O×Rd double mutants do not show differences in pupillary size between light and dark. As it does not have any type of photoreceptor cell, the signaling pathway that controls the muscles of the iris cannot be activated, so it maintains a constant area whether they are illuminated or not.

The optomotor test measures the visual acuity of the animal by varying the spatial frequency, direction, and contrast of the stimuli [44,45,46,47]. This test involves the entire circuit of the retina. Thus, the absence of any cellular component alters the animals’ visual acuity and, therefore, their ability to detect the different stimuli used [48,49,50]. The optomotor test showed that there was a model (*Opn4^−/−^*) in which there was no variation in visual acuity with respect to the control, while in the other two (Rd10 and *Opn4^−/−^* × Rd10), visual acuity had been lost due to the degeneration of classical photoreceptors, responsible for the visual imaging pathway.

The rd retinal degeneration models, and specifically Rd10, have been characterized electroretinographically (ERG) [14,51,52,53,54]. These models experience a gradual loss of amplitude of the components of the different ERG waves as degeneration progresses. To ensure the complete loss of rods and cones, animals older than 60 days postnatal were chosen, verifying that there was an absolute disappearance of ERG waves, both in the Rd10 models and in the O×Rd double mutants.

The pERG was performed to know the functional state of the inner layers of the retina by recording the response to a visual grating stimulus with constant luminosity, which results in a standard ERG wave suppression [55,56]. In rd type retinal degeneration, the structural loss of the outer retina in the Rd10 model causes the disappearance of the three characteristic components of pERG waves (N35, P50 and N95), present in C57 mice, even when the inner retina remains structurally stable.

From the retina, the axons of the optic nerve project to different nuclei of the central nervous system [57,58,59,60]. The final target of this pathway is the primary visual cortex. The way to characterize the projection pathways to the central nervous system is through visual evoked potentials. One of the hypotheses that had to be discarded when making the O×Rd model was that there was some signal from photosensitive elements remaining in the retina that could not have been recorded in the ERGs due to their extremely low amplitude. The results showed that C57 animals presented robust VEP signals, similar to those obtained in other studies [61,62]. However, the O×Rd double mutant model was unable to evoke any kind of response from the visual cortex.

The structural study was carried out using immunohistochemistry and DNA inclusion markers. The results obtained corroborated the data shown above and those of previous studies [14,51,52,53,54].

In the models in which the classic photoreceptors (Rd10 and O×Rd) were lost, the outer retina completely disappeared, as evidenced by the absence of DAPI labeling in the outer nuclear layer. The specific loss of cones and outer segments of the rods was determined by labeling with specific antibodies against cone arrestin and rhodopsin, proteins expressed in the cones and outer segments of the rods, respectively. This loss impaired visual function, justifying the absence of signals registered in the different functional tests. Despite this, structurally, these models (Rd10 and O×Rd) do not show any abnormality in cell distribution or integrity in the inner retina, as observed by specific labelings in bipolar and ganglion cells (PKCα and Brn3a antibodies, respectively). Therefore, the structure of the inner retina remains intact, but unable to respond to light stimuli, due to the loss of synaptic transmission from the photoreceptors. This does not rule out the possibility that, at advanced ages, structural damage to the inner retina may occur.

To label the intrinsically photosensitive RGCs, a specific antibody against melanopsin was used, which is expressed in a small population of specific ganglion cells (approximately 2% [11,63]). The O×Rd and *Opn4^−/−^* double mutant models showed absence of labeling for melanopsin. On the contrary, the Rd10 model showed a positive melanopsin labeling, which explained the strong rejection of light spaces and the intense pupillary contraction when exposed to light (light/dark transition and pupillary reflex test, respectively). Meanwhile, the O×Rd animal model, according to the absence of melanopsin, did not present this type of behavior or reflexes.

In summary, the O×Rd model was not able to evoke any type of electroretinographic signal, despite the fact that the structure of the internal retina and the projection pathways to the central nervous system remained intact. Therefore, this model is suitable for conducting optopharmacological, optokinetic, and behavioral studies since it does not present any interference from a residual visual capacity that could falsify the results.

Although the O×Rd blindness animal model is incapable of detecting and responding to light stimuli, it has some disadvantages coming from the Rd10 animal model that may lead to certain experimental limitations.

Beyond the degeneration that completely destroys the outer retina in Rd10, there is another degenerative process that could cause certain complications in an experimental study. The disappearance of the rods means that the bipolar cells that contacted them retract their dendritic tree until they disappear, maintaining their cellular structure. At 25 days postnatally, the dendrites of the bipolar rod cells begin to retract, disappearing by around 45 days of age. As the cones degenerate later, the retraction of the dendrites of the bipolar cone cells is also later, at 4 or 5 months of life. The disappearance of these dendritic trees implies the loss of a large number of postsynaptic receptors, such as the glutamatergic metabotropic glutamatergic receptor mGluR6 [14,51,53,54,64]. This could be a problem for new treatments or physicochemical approaches targeting this or other types of receptors or proteins expressed in its dendritic tree. However, despite this dendritic regression, both the cell body and axon terminations of bipolar cells remain intact. 

Another limitation to take into account is that while the mutation in the *Opn4* gene inhibits the synthesis of melanopsin from the embryonic state, the degeneration of the rods and cones begins after approximately 16 postnatal days. However, the time at which degeneration is complete varies greatly between individuals, which may present difficulties when performing some electroretinographic techniques in very young subjects [54].

Finally, with age, the vascular density of the different retinal plexuses decreases. In addition, several studies have shown a more marked decrease in vascular density in Rd10 animals due to photoreceptor degeneration [65,66,67]. Likewise, in retinal neurodegeneration, such as in retinitis pigmentosa, a decrease in blood flow has been observed [68,69,70]. Despite the drawbacks presented, the choice of a genetic model avoids the need for the researcher to induce physicochemical or surgical damage. In addition, other administration methods of toxic compounds could cause systemic alterations.

Currently, there are a large number of animal models in which, through genetic mutations, some of the photosensitive elements of the retina are absent. However, only one other genetic animal model has been found in which the response of the three photosensitive elements of the retina is absent. It is a homozygous triple knockout (TKO) animal model for the *Rho^−/−^*, *Cnga3^−/−^* and *Opn4^−/−^* genes, generated through the crossing of double knockouts *Cnga3^−/−^* and *Rho^−/−^* with *Opn4^−/−^* animals. This model has been used to test the efficacy of a new photochromic AMPA agonist (photoswitch) [71].

Rhodopsin is involved in the rod phototransduction cascade, and it also has a structural role in their outer segments. The absence of rhodopsin expression (*Rho^−/−^*) will disrupt phototransduction in the rods and result, over time, in their structural collapse [72].

On the other hand, the *Cnga3^−/−^* mutation generates a truncated CNGA3 subunit, which impairs the function of cyclic nucleotide-activated receptors in cones, so they will not present any type of response, suffering a progressive degeneration [72,73].

Unlike the O×Rd model, the degeneration suffered by the TKO model runs much more slowly [74,75]. Thus, the retina of these animals shows a complete structure during at least 30 postnatal days, while the O×Rd model suffers major degeneration between 20–25 days, completing at 60 days postnatal [54].

Therefore, the O×Rd animal model opens a more efficient time window to carry out therapeutic studies of new ophthalmological approaches against retinal degeneration. In this way, the O×Rd animal model has been used successfully in the test of a new operational photoswitch model [74]. A photochromic ligand associated to an adrenergic modulator (adrenoswitches) was used as an agonist of α1-adrenergic receptors present in the iris musculature. These receptors control the processes of mydriasis and miosis, depending on the incident light. The adrenoswitch was administered topically, and after a short period of time, stimulation with UV light caused mydriasis, while animals administered with vehicle did not show any type of pupillary contraction. When the stimulation ended, the effect quickly reversed. Thus, it was shown that the binding of the adrenergic modulator with α1-adrenergic receptors was selectively controlled by illumination through specific wavelengths.

Therefore, only an animal model such as O×Rd, which shows a total loss of the photosensitive elements of the retina, could be used satisfactorily to carry out this study. The use of other models, such as those that only presented rod degeneration or did not present retinal degeneration, would have posed a problem for the detection of adrenoswitch activity.

The importance of the O×Rd model lies in the fact that it allows for determining the efficacy of therapeutic strategies that need to be carried out in conditions of absolute blindness, that is, without any remaining photosensitivity that falsifies the results. One example of such strategies is photoswitch technology.

## 4. Materials and Methods

### 4.1. Animal Housing Conditions

All procedures were carried out according to European (Directive 86/609/CEE) and national (Royal Decree 53/2013, February 1st) regulations in force on the protection of animals that are used for experimentation and other scientific purposes, and Law 32/2007 for the care of animals in their exploitation, transport, experimentation, and sacrifice; in addition, the guidelines of the association for research in vision and ophthalmology were followed (the Association for Research in Vision and Ophthalmology, ARVO). All experiments were approved by the Ethics Committee of the University of Alcalá for the use of experimental animals (CEI-UAH-AN2016008//PROEX 147 y CEI-UAH-AN2021009 PROEX 147).

The mice were housed in ventilated racks with ultrafiltration cages of air through HEPA filters (high-efficiency particulate air filters) with a ventilation rate of more than 30 atmospheric changes per hour. The room had an ambient temperature of 21 ± 1 °C and a relative humidity of 55 ± 10%. The animals were fed A04 feed pellets (Panlab SL, Spain) and allowed to eat and drink ad libitum. The bed of the cages was made of absorbent sawdust. Weekly cleanings were performed. Circadian cycles were established with an alternation of 12:12 h of light/dark and with an average of 20 Luxes in each cage during the day phase and total darkness during the night phase.

As controls, C57Bl/6J animals (*n* = 8) (C57 or WT); C57Bl/6J animals with a deletion mutation of the *Opn4^−/−^* (Opn4) gene (*n* = 8); and animals showing a mutation in the Pde6b gene encoding a truncated phosphodiesterase subunit, known as *Pde6b^rd10/rd10^* (Rd10) (*n* = 8) with an age of 100 postnatal days, were used. Characterization was carried out in a group of eight double mutant animals *Opn4^−/−^* × *Pde6b^rd10/rd10^* (O×Rd) raised in the laboratory, with an age of 100 postnatal days.

### 4.2. Anaesthetic Procedures

The anesthesia protocol was applied to all the experiments carried out except in the records of the pupillary reflex. First, the animals were weighed and anesthetized using a ketamine anesthetic solution (Ketamidor, Richter Pharma AG, Wels, Austria) (100 mg/mL), xylazine (Xilagesic, CALIER, Barcelona, Spain) (20 mg/mL), and NaCl (Grifols, Barcelona, Spain) (0.9%), at a final concentration of 0.5mL/150g, administered intraperitoneally with a 25G needle.

In the pupillary reflex studies, the animal was anesthetized with isoflurane (gas) (2%, Isoflo©, Zoetis S.L., Madrid, Spain). A TEC 3 vaporizer was used (MSS International Ltd., Keighley, UK), maintaining a constant flow of 0.4 L/min of O^2^.

After completing the procedures that required anesthesia, the animals were allowed to rest in a cage, placed on a thermal blanket to promote their recovery.

### 4.3. Surgical Procedures for the Implantation of a Chronic Electrode

In order to carry out the double electrophysiological recording of electroretinogram and visual evoked potentials (VEP), it was necessary to carry out chronic implantation of electrodes in the visual cortex of the animals, based on the intervention carried out in other studies [75,76,77,78]. All surgical material was previously disinfected with 70% ethanol. The animals were weighed, anesthetized, and placed in a stereotaxic apparatus (Stoelting, Wood Dale, IL, USA). Hair was removed from the area between the ears, and the entire surface was properly cleaned and disinfected. By means of a longitudinal cut right in the center of the disinfected area, between both ears, the skull was exposed, and the connective tissue was removed by scraping it with the scalpel. A single electrode was placed on the left visual cortex of the animal (V1, 0.61 mm rostral from Lambda and 2.3 mm left lateral). A hole was made in the corresponding place by using a hand drill and a stainless-steel drill bit (0.8 mm diameter) (Dremel Multipro, Madrid, Spain). A stainless-steel screw (M1-4mm) was placed in the hole, allowing the base to come into contact with the surface of the brain without entering the visual cortex. Finally, the edges of the wounds were brought into contact to promote healing, and the area was covered with dental resin (DuraLay, Reliance, IL, USA) to protect and promote the correct fastening of the screw. Pain reliever (Meloxidyl, Veterinaria Esteve, Barcelona, Spain) was administered, dissolved in water at a dose of 5 mg/100 g/day. Any type of manipulation was avoided until 48 h after the intervention, to ensure the correct recovery of the animal. During this time, the animals were found to recover satisfactorily and did not show any signs of pain or agony.

### 4.4. Light/Dark Transition Test

To carry out this test, a box was constructed with two compartments 59 cm high and 28.5 cm on the side, one being white and the other dark. These compartments were separated by a small opening in the wall, which was blocked to prevent the flight reflex of the animals when they were introduced [37]. In the white box, a strip of LEDs was placed (Ledflexi Inspire, Leroy Merlin, France). The light intensity in the white box was 484.3 Lux, while in the black box, the light intensity was 0.21 Lux. The animals were introduced into the box and after a few seconds the passage between compartments was unlocked, allowing the animal to begin to explore freely. The time that each animal spent in the compartments was timed, for a period of 5 min. Compartment change was defined by the animal passing completely through the opening. The procedure was repeated three times for each of the animals.

### 4.5. Optomotor Test

The equipment was made up of four screens (FLATRON, LG, Seoul, South Korea) facing each other to form a closed enclosure. The animals were introduced in the center of the enclosure, inside a transparent methacrylate cylinder, which restricted their movements, favoring the concentration of the animal in the stimulus. The entire cubicle was insulated from the outside. A closed-circuit infrared camera (AVC-D5CE, SONY, Tokyo, Japan) was inserted through a small opening in the upper part of the enclosure, which allowed the animal to be monitored.

The screens showed a series of moving black and white bars (sinusoidal cycle), which present different spatial frequencies (0.011, 0.022, 0.044, 0.088, 0.177, and 0.355 cpd), as well as different contrasts (100, 50, 25, 10, and 5%). In this way, the bars generated a pseudo-rotating effect that the animals could follow with a head movement. The stimuli were presented for 20 s in both directions of rotation (clockwise and anti-clockwise) in a random way, thus allowing the study to present greater objectivity. After presentation of the stimulus, the operator assigned an affirmative value if the animal had moved its head in the same direction as that indicated by the stimulus presentation software. A criterion of three hits per stimulus was applied to ensure the veracity of the results.

### 4.6. Pupillary Light Reflex (PLR)

During the procedure, the mice were kept anesthetized by isoflurane, as described in the anesthesia section. The animal was placed on a water closed-circuit heating blanket (T/Pump TPP522, Gaymar Industries, Orchard Park, NY USA) to maintain body temperature, allowing resting for 5 min to stabilize the pupil. Subsequently, a light stimulus was applied for one minute, followed by a 2-min dark cycle so that the pupil returned to the basal state. This procedure was carried out three times in a row, in each animal. Finally, the animal was allowed to recover on a thermal blanket, and after a few minutes, it was returned to its corresponding cage. To study the variation of the pupillary area when presenting the light stimulus, an infrared camera (AVC-D5CE, SONY, Tokyo, Japan) coupled to a laboratory magnifying glass (Wild Heerbrugg, Leica, Switzerland) focused at 15 cm was used with the objective, which allowed for photography of the pupil of the animals every 10 s. Subsequently, the pupil area was measured using the ImageJ image analysis software (U. S. National Institutes of Health, Bethesda, MD, USA) in its portable version (FIJI).

### 4.7. Full-Field Electroretinogram

The animals were kept in the dark for 12 h prior to anesthesia, which was carried out, as indicated above, under dim red light, the recordings being carried out in complete darkness. The animals were placed inside a Faraday box, on a heated water flow blanket (T/Pump TPP522, Gaymar Industries, NY, USA) to keep the body temperature constant at 37 °C, while the pupils were dilated by applying a topical drop of 1% tropicamide (Alcon Cusí, SA, El Masnou, Barcelona, Spain). A needle was placed at the base of the tail for grounding and a reference electrode was placed on the tongue. For the recording, a gold band electrode placed on the cornea was used. A few drops of methylcellulose were applied (2% Methocel, Omnivision, Neuhausen, Switzerland), to protect the cornea and improve conductivity. The signals were recorded in the right eye in response to stimulation produced by a Gandfel bell. A first scotopic phase was performed with flashes of increasing intensity (−4.0, −3.0, −2.0, −1.5, −1.0, −0.5, −0.0, 0.5, 1.0, and 1.5 log cd·s^−1^·m^−2^) and at least 10 signals per stimulus were averaged, with increasing interstimulus times, to avoid photoreceptor bleaching. The signals were amplified 1000 times by means of a Grass amplifier (CP511 AC amplifier, Grass Instruments, Quincy, Massachusetts, USA) and were filtered with a band pass established between 0.3 and 1000 Hz. To record the oscillatory potentials, the pass filters band were changed at 30 and 10,000 Hz, using an intensity of 1.5 log cd·m^−2^. The electrophysiological signal was digitized at 10 kHz with a Power Lab 4/35 data acquisition card (ADInstruments Ltd., Oxfordshire, UK) and a minimum average of 10 signals. For the photopic recordings, the animals were adapted to light with an intensity of 30 cd·m^−2^ for 5 min, and flashes of increasing intensities were applied (−1.0, −0.5, −0.0, 0.5, 1.0, and 1.5 log cd·s·m^−2^), at interstimulus times lower than those of the photopic record. The flicker response was recorded at a frequency of 20 Hz and intensity of 1.5 log cd cd·s·m^−2^. All the amplitudes of the different components of the ERG waves were measured manually using the commercial software LabChart Pro v.8.1.13 (ADInstruments Ltd., Oxfordshire, UK).

### 4.8. Pattern Electroretinogram

The animals were anesthetized and placed on a water flow thermal blanket (T/Pump TPP522, Gaymar Industries, Orchard Park, NY, USA) inside a Faraday cage. On this occasion, the pupils were not dilated, to improve the visualization of the stimulus. The various electrodes were placed using the same configuration as in the full-field electroretinogram, and the responses of the right eye to the stimuli were recorded. Stimulation equipment (Roland Consult, Brandenburg, Germany) connected to a screen (919Pz, AOC, Taipei, Taiwan) was used to present the stimuli; the screen was located 25 cm from the animal’s head.

The stimulation area consisted of a black-and-white checkerboard (99% contrast), alternating at a frequency of 1 Hz and configured with various spatial frequencies (0.03, 0.06, 0.08, and 0.12 cpd). Prior to the presentation of the stimuli, it was verified that the impedance of the electrodes was less than 10 KΩ. The signals were amplified 10,000 times and filtered between 0.1 and 300 Hz. An average of 400 signals was performed in each stimulus. The amplitude of the waves was measured manually from the positive component of the wave (P50) to the negative component (N95), using the stimulator’s own software.

### 4.9. Visual Evoked Potentials (VEP)

Approximately one week after electrode implantation, the animals were anesthetized following the previously discussed ketamine/xylazine protocol, and placed on a thermal water flow blanket (T/Pump TPP522, Gaymar Industries, Orchard Park, NY, USA) to keep the body temperature constant, all inside a Faraday box.

Unlike other studies, which implant reference electrodes in the prefrontal cortex [34,35,36,37], in our study we used, as a reference electrode, a gold reed placed on the tongue of the animals, in a similar way to what was performed for the electroretinographic techniques. In this way, the surgery was less damaging, since the need to implant a second chronic electrode was avoided, but it allowed obtaining solid records of the different components of the VEP. For grounding, a needle was inserted at the base of the tail, while for the VEP registration, a fine clamp was used, connected to the electrode surgically implanted in the skull of the animal.

To perform the recording, the same protocol discussed above was used for ERG recordings, with a properly calibrated Gandfeld bell. Approximately 300 averages were performed. The VEP signals were amplified 100,000 times and filtered through a band pass filter set at a frequency between 5 and 500 Hz, using a NeuroLog System amplification and filter equipment (NL900D NeuroLog System Rac, Digitamer Ltd., Letchworth Garden City, UK). The electrophysiological signal was digitized at 10 KHz with a Power Lab 4/30 data acquisition card (ADInstruments Ltd., Oxfordshire, UK). The components of the waves were analyzed manually using the commercial software LabChart Pro v.8.1.13 (ADInstruments Ltd., Oxfordshire, UK).

### 4.10. Inmunohistochemistry

The mice were sacrificed by overdose of pentobarbital (Dolethal, Picemar, Castellón, Spain), and the eyes were rapidly enucleated and fixed with 4% PFA for 1 h and 30 min. After that time, the lens was removed and kept for a further 30 min in 4% PFA, to ensure correct fixation. Subsequently, to cut them into the cryostat, the eyes were processed using an increasing sucrose gradient (20–30% sucrose (*w*/*v*) in 0.1 M phosphate buffer, pH 7.4) and kept at 40% sucrose overnight. The following morning, they were included by OCT (Optimal Cutting Temperature, Sakura Finetek, CA, USA) and frozen at −20 °C by cryostat (Leica CM1950, Leica, Switzerland). Serial 15 µm cross sections were made with the cryostat. After leaving the sections to stand, three washes of 10 min each were carried out, with shaking. A preincubation with 0.5% Triton X-100 in 0.1M PBS was carried out to block nonspecific signals in the tissues. The sections were incubated with 0.5% Triton X-100 in 0.1M PBS, 2% serum, and the primary antibodies (see concentration in Table 1) overnight in a humid chamber. After primary incubation, three washes of 10 min each were carried out, followed by incubation with the secondary antibodies, (see Table 2), diluted in 0.5% Triton X-100, 0.1M PBS and 2% serum, for 1 h and 30 min. Afterwards, three new washes of 10 min each were carried out, and a DNA intercalator marker (4′, 6-diamidino-2-phenylindole (DAPI)) (SIGMA) was added at a concentration of 0.01% to determine the nuclear layers of the retina. This chromophore emits at 470 nm. Finally, three new washes were carried out and the sections were mounted with the appropriate mounting medium.

### 4.11. Statistical Analysis

Statistical analysis was carried out using the software GraphPad Prism 8.0 (GraphPad Software Inc., La Jolla, CA, USA). To check the normality between groups, the Shapir–Wilk test was used. For comparisons between two groups, paired or unpaired *t*-test was carried out depending on the characteristics of each comparison. For the analysis of several groups with more than two variables, two-way ANOVA and Sidak’s multiple comparison were used. In all cases, the value of statistical significance was established as *p*-value < 0.05. Statistical significance is indicated on each of the images by asterisks (*, *p* < 0.01; **, *p* < 0.005; ***, *p* < 0.001; ns, non significance).

## 5. Conclusions

The double mutant animal model *Opn4^−/−^* × Rd10, obtained from crosses between carriers of a mutation in the *Pde6b* gene and in the *Opn4^−/−^* gene, has been thoroughly characterized as a model of absolute blindness.

The mutation in the *Pde6b* gene causes degeneration of rods and cones, while the knockout in the *Opn4^−^* gene prevents the synthesis of melanopsin, expressed in intrinsically photosensitive ganglion cells. Combination of both mutations provides us with an animal without any photosensitive element in the retina, but which maintains its internal structure, making it an ideal model for the study of new molecules or therapies, such as for optogenetics or optopharmacology.

## Figures and Tables

**Table 1:** Primary antibodies used in the labeling of the different cell types of the retina.

Primary Antibodies Table					
Primary Antibodies	Type	Host	Concentration	Provider	Labelling
Anti-Cone Arrestin	Polyclonal	Rabbit	1:10,000	Merk-Millipore	Cones
Anti-Calbindin	Polyclonal	Rabbit	1:1000	SIGMA	Photoreceptors
Anti-Melanopsin	Polyclonal	Rabbit	1:100	ATSbio	ipRGCs
Anti Rhodopsin	Monoclonal	Mouse	1:200	Merk-Millipore	Rod Outer Segments
Anti-PKCα	Polyclonal	Rabbit	1:1000	SIGMA	Bipolar Cells
Anti-Brn3	Polyclonal	Goat	1:200	Quimigen (Santa Cruz)	Retinal Ganglion Cells

**Table 2:** Secondary antibodies for the detection of the different primary antibodies used in immunohistological labeling.

Secondary Antibodies Table					
Secondary Antibodies	Fluorochrome	Host	Concentration	Provider	Color
Rabbit Anti-IgG	Cy^TM^2	Donkey	1:200	VITRO (Jackson)	Green
Rabbit Anti-IgG	Cy^TM^3	Donkey	1:200	VITRO (Jackson)	Red
Mouse Anti-IgG	Cy^TM^2	Donkey	1:700	VITRO (Jackson)	Green
Goat Anti-IgG	Cy^TM^5	Donkey	1:500	VITRO (Jackson)	Infrared

## Data Availability

Not applicable.
